# Déficit combiné en facteurs V et VIII de la coagulation: à propos d´une fratrie de trois cas

**DOI:** 10.11604/pamj.2021.39.65.24559

**Published:** 2021-05-21

**Authors:** Hassane Mamad, Souad Benkirane, Yousra El Aissaoui, Zakia Berchane, Azlarab Masrar

**Affiliations:** 1 Laboratoire d'Hématologie, Équipe de Recherche en Hématologie, Faculté de Médecine et de Pharmacie, Université Mohammed V, Rabat, Maroc,; 2 Laboratoire Central d´Hématologie, Centre Hospitalier Universitaire Ibn Sina, Rabat, Maroc

**Keywords:** Hémostase, hémorragie, DF5F8, LMAN1, MCFD2, rapport de cas, Hemostasis, hemorrhage, DF5F8, LMAN1, MCFD2, case report

## Abstract

Le déficit combiné en facteurs V et VIII de la coagulation (DF5F8) est un désordre constitutionnel de transmission autosomique récessif. C´est une famille de quatre enfants, issus de consanguinité. La fille aînée adressée pour exploration d´allongement du Temps de Céphaline avec activateur et du Temps de Quick, associé à des manifestations hémorragiques. Le dosage des facteurs de coagulation montre un déficit combiné en facteurs V et VIII, et taux normaux des autres facteurs de coagulation. On trouve un DF5F8 chez deux filles et un garçon. Deux gènes codent pour protéines LMAN1 (Lectin MANnose-Binding1) et MCFD2 (Multiple Coagulation factor deficiency2), sont impliquées dans le passage intracellulaire des FV et VIII, dont certaines mutations provoquent un déficit combiné en facteur V et VIII. Diagnostic du DF5F8 est possible en routine surtout chez des patients issus de consanguinité avec un contexte clinico-biologique évocateur.

## Abstract (en)

Combined deficiency of clotting factor V and factor VIII (DF5F8) is a congenital autosomal recessive disorder. This study involved a family of four children born to consanguineous parents. The eldest daughter was referred for assessment of activated partial thromboplastin time and prothrombin time associated with hemorrhagic manifestations. Coagulation factor dosing showed combined deficiency of factor V and factor VIII as well as normal levels of other coagulation factors. DF5F8 was detected in two girls and a boy. Two protein coding genes LMAN1 (lectin, mannose binding 1) and MCFD2 (multiple coagulation factor deficiency2) were involved in the intracellular passage of Factor V and Factor VIII, including some mutations which caused deficiency of Factor V and VIII. The diagnosis of DF5F8 is routinely possible, especially in patients born to consanguineous parents with a suggestive clinico-biological condition.

## Introduction

Le déficit combiné en facteurs V et VIII de la coagulation (DF5F8) est un désordre hématologique constitutionnel, décrit depuis 1954, par Oeri *et al*. [1]. Ce trouble hématologique est rare, de transmission autosomique récessif, et dont la prévalence est estimée entre 1/100.000 à 1/1.000.000, représentant la forme la plus fréquente d´anomalie constitutionnelle, associant plus d´un facteur de coagulation [2], et n´étant pas lié à la coïncidence accidentelle de plusieurs déficits héréditaires génétiquement distincts, mais lié à une anomalie génétique unique [2,3]. Nous rapportons trois cas de DF5F8 chez une même famille, diagnostiqués au Laboratoire Central d´Hématologie Ibn Sina Rabat, Maroc.

## Patient et observation

### Informations des patients

**Figure 1:**
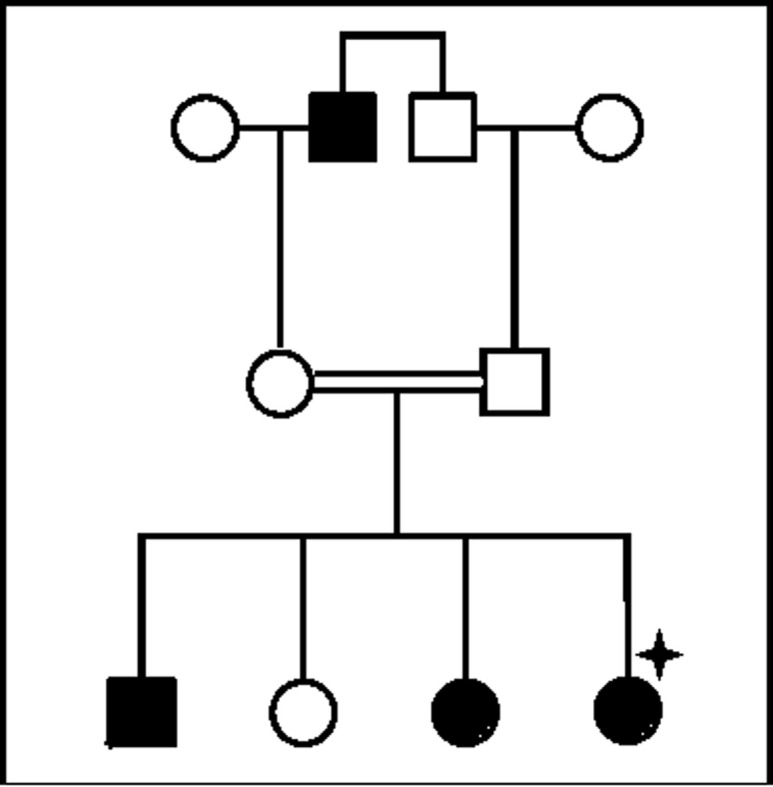
analyse généalogique (pédigrée des sujets F5F8D); pointe d´étoile noir = proband (personne atteinte de la maladie génétique à partir de laquelle on fait le conseil génétique), noir = sujets avec antécédents de saignements cliniques, blanc = sujets normaux

L´étude a été faite sur un échantillon de 6 personnes d´une même famille. Il s´agit d´une famille de quatre enfants originaires de Khemissat (Ouest du Maroc): trois filles et un garçon, âgés respectivement de 11, 9, 7 et 4 ans, issus de parents consanguins de deuxième degré (Figure 1). La découverte de cette anomalie a été faite chez la fille aînée adressée au laboratoire pour exploration d´un allongement du temps de céphaline avec activateur (TCA) et d´une baisse du taux de prothrombine (TP), associé à des manifestations hémorragiques massives lors d´une extraction dentaire et des tâches ecchymotiques après un traumatisme. La fiche d'exploitation dument renseignée par le clinicien et complétée par le laboratoire contient les informations suivantes: les caractéristiques épidémiologiques: nom, prénom, âge; l'histoire hémorragique: personnels (mode, fréquence et intensité de saignement) et familiaux (consanguinité) (Tableau 1).

**Tableau 1:** données épidémiologiques et cliniques de la famille étudiée

Sujet	Age (ans)	Sexe	Consanguinité	Renseignements cliniques
Père	38	M	2ème degré	Pas d´histoire hémorragique
Mère	38	F		Pas d´histoire hémorragique (Mais son père présente des épistaxis épisodiques)
Sœur 1	11	F	Fratrie	Gingivorragie après extraction dentaire, taches ecchymotiques après traumatisme
Sœur 2	9	F		Gingivorragies et épistaxis
Sœur 3	7	F		Pas d´histoire hémorragique
Frère	4	M		Hémorragies minimes lors de la circoncision

M: Masculin, F: Féminin

### Évaluation diagnostique

Au sein du laboratoire, les tests d´hémostase sont réalisés par la méthode optique sur l´automate Sysmex CS-2500 et les réactifs Siemens (Tableau 2) et l´hémogramme sur l´automate Sysmex XN9000. La réalisation des bilans d´hémostase chez la fille aînée, a confirmé l´allongement du TCA, rapport TCA (TCA patient / TCA témoin) à 2.52 (Ratio normal = 1.2) et un TP bas à 45% (TP normal entre 70 et 100%), ainsi que le dosage spécifique des facteurs de la coagulation montre un déficit combiné en facteurs V et VIII à 10% et 12.6% respectivement, avec des taux normaux des autres facteurs de coagulation et un indice de Rosner < 12%. L´hémogramme sans particularités.

**Tableau 2:** résultats des tests d´hémostase réalisés

	Père	Mère	Sœur 1	Sœur 2	Sœur 3	Frère
TP (%)	98	100	45	52	94	49
TCA (Ratio)	1.07	1.05	2.52	2.16	1.2	2.34
IR (Indice Rosner %)	0	0	11.6	10.3	0	6.6
FV (%)	109.4	105	10	14.7	124.1	20.2
FVIII (%)	158.6	127.8	12.6	18.6	152.4	21.2
vWF-Ac (%)	123.9	95.7	72.5	59.3	145.9	123.4
Fibrinogène (g/l)	2.4	3.4	2.6	2.5	2.1	2.2
FII (%)	104.4	116.5	96.9	101.2	89	103.7
FVII (%)	82	115.1	82	85.8	72.8	90.9
FIX (%)	95.1	175.4	71.5	83.1	69.2	100.1
FX (%)	87.2	94.9	87.2	82.5	82.5	100.7
FXI (%)	106.4	100.5	56.6	66	90.8	84
FXII (%)	62.7	81.6	103.5	54.4	40	76.2

TP: Taux de prothrombine, TCA: temps de céphaline avec activateur, IR: Indice Rosner, FII: Facteur II de la coagulation, FV: Facteur V de la coagulation, FVII: Facteur VII de la coagulation, FVIII : Facteur VIII de la coagulation, FIX : Facteur IX de la coagulation, FX: Facteur X de la coagulation, FXI: Facteur XI de la coagulation, FXII: Facteur XII de la coagulation. vWF-Ac: Activité fonctionnelle en facteur de von Willebrand

### Suivi et résultats

Après la mise du diagnostic définitif du déficit combiné en facteurs V et VIII de la coagulation, la patiente a été adressée et invitée aux visites régulières au service d´hématologie pédiatrique. Par ailleurs, la présence d´un syndrome hémorragique dans la fratrie nous a amené à élargir nos investigations à toute la fratrie et aux deux parents à la recherche de cas similaires (Tableau 1, Tableau 2). Au total, on trouve un DF5F8 chez deux filles et un garçon et des bilans normaux chez les parents et la fille de 7 ans (avec un déficit noté en facteur XII de la coagulation à 40%). Ces résultats ont été confirmés sur des contrôles réguliers ce qui a permis de conclure un DF5F8.

## Discussion

Le DF5F8 est une maladie héréditaire de la coagulation qui est extrêmement rare, (1/1.000.000) dans la population générale [4]. Donnant un trouble hémorragique récessif autosomique, avec des taux plasmatiques des facteurs de coagulation FV et FVIII, réduits à 5-30% des niveaux normaux [5]. Notre étude présente une maladie génétique qui reste rare et peu diagnostiquée vu sa confusion avec le diagnostic d´une hémophilie A mineure ou un déficit congénital en FV de la coagulation surtout chez des sujets féminins atteints de ce déficit. Deux gènes codent pour deux protéines LMAN1 (Lectin MANnose-Binding1), situé sur le chromosome 18q21 et MCFD2 (Multiple Coagulation Factor Deficiency 2), situé sur le chromosome 2p21. Ces deux protéines en formant un complexe stœchiométrique 1: 1, sont impliquées dans le pliage et le transport intracellulaire des FV et VIII entre le réticulum endoplasmique et l´appareil de Golgi avant la sécrétion [6], dont certaines mutations décrites entravent la structure des molécules chaperonnes et provoquent un déficit combiné en FV (1 à 33%) et FVIII (1 à 31%) de la coagulation donnant un syndrome hémorragique variable et de sévérité dépendante surtout du taux de FVIII avec une symptomatologie similaire à celle de l´hémophilie mineure. Environ 60 à 80% des patients atteints de DF5F8 présentent des saignements prolongés à la suite d'une blessure ou d'une intervention chirurgicale. Des saignements gingivaux et des épistaxis surviennent chez plus de 50% des patients, les hémarthroses, typiques de l'hémophilie A et B, surviennent chez moins d'un tiers des patients. La majorité des patients sont d'origine moyen-orientale ou indienne [7].

On note que le diagnostic moléculaire reste indispensable pour identifier les désordres précis sur les gènes LMAN1 et MCFD2 [8,9]. Sur le volet thérapeutique, le traitement des épisodes hémorragiques ou de la prophylaxie chirurgicale chez les patients atteints de DF5F8 a été le plus souvent, la perfusion du plasma, qui fournit à la fois les facteurs V et VIII. Par ailleurs des données limitées ont été publiées, décrivant l´utilisation de la desmopressine intranasale (DDAVP) chez leurs patients atteints du DF5F8 [7]. Notre étude présente certaines limites (absence de ces tests dans notre institution), telles que la recherche des mutations responsables des désordres précis sur les gènes LMAN1 et MCFD2.

## Conclusion

Le diagnostic du DF5F8 est possible en routine surtout chez des patients issus de consanguinité et présentant un contexte clinico-biologique évocateur. Néanmoins cette maladie reste très peu diagnostiquée, soit par l´absence de signes cliniques graves induisant une absence de consultation médicale, soit par un diagnostic d´hémophilie A mineure dont le TP est légèrement bas par excès chez les sujets masculins atteints de ce déficit. Pour cette raison un dosage du FV doit être réalisé pour tout déficit modéré en FVIII, comme nous recommandons l'évaluation du niveau de facteur VIII de coagulation chez les patients présentant un allongement du TCA, une baisse du TP et un déficit en facteur V de la coagulation.
